# Advanced Care Plan Among Older Chinese in Residential Aged Care: A Retrospective Review

**DOI:** 10.1177/10436596241296817

**Published:** 2024-11-13

**Authors:** Minah Amor Gaviola, Simon Pedzisi, Kerry Jill Inder, Amanda Johnson

**Affiliations:** 1 The University of Newcastle, Callaghan, New South Wales, Australia; 2 Opal HealthCare, Sydney, New South Wales, Australia; 3 Hunter Medical Research Institute, New Lambton Heights, New South Wales, Australia

**Keywords:** advanced care plan, cultural groups, culture, diversity, palliative care, nursing home, older people

## Abstract

**Introduction:**

Worldwide in the population of older people, ethnic diversity is prevalent and therefore warrants culturally sensitive advanced care planning. This study aimed to explore advanced care planning documentation related to the cultural needs of residents of Chinese ethnicity in Australian aged care facilities.

**Methods:**

A retrospective review of advanced care plan documentation was undertaken among 31 older Chinese residents with life-limiting illnesses across two residential aged care facilities in New South Wales, Australia. Data were analyzed using descriptive statistics.

**Results:**

90% of residents had advanced care planning documentation. The presence of the resident and their representative and medical care directives were well documented. Specific details on the provision of palliative care that considers the person’s cultural needs and preferences were limited.

**Discussion:**

Findings suggest the need for further research that explores an optimal way of embedding culture-specific information and the development of a culturally sensitive advanced care plan for people of Chinese ethnicity.

## Introduction

Worldwide migration is continuously increasing and, as such, provides ethnic and cultural diversity among many populations (International Organization for Migration, 2022) including the population of Australian older people (Australian Institute of Health and Welfare [AIHW], 2023a). With the prevalence of chronic and life-limiting illnesses in older age, a growing demand for palliative care worldwide is expected (United Nations Department of Economic and Social Affairs, 2023; World Health Organization, 2020). Similar to other Western countries such as the United States (Lima & Miller, 2018) and Europe (Smets et al., 2018), Australian residential aged care facilities (RACFs) (equivalent to nursing homes and long-term care facilities) have realized an exponential growth in the provision of palliative care (AIHW, 2022).

Palliative care is defined by the World Health Organization as “an approach that improves the quality of life of patients and their families who are facing the problems of life-threatening illnesses” (World Health Organization, 2002, p. 84). With a broader definition of palliative care embracing noncancer diagnoses (World Health Organization, 2020) and a greater presence of multimorbidity among older people (Chowdhury et al., 2023), the care delivered in RACFs (or equivalent) has significantly changed to meet the needs of the older person with high acuity and frailty (AIHW, 2023c).

In Western countries, people of Chinese ethnicity are among the largest group of migrants (International Organization for Migration, 2022). With the traditionally valued filial piety, families play a significant role in the care of older Chinese people (Lu et al., 2021). Even with societal and economic changes impacting Chinese cultural traditions, family-based care of older Chinese people is still strongly expected and preferred over placement into an aged care institution (Lu et al., 2021; Ma et al., 2023). However, despite lesser preference toward this (Cheng et al., 2023; Ma et al., 2023), the literature shows that older people of Chinese ethnicity with life-limiting illness in Western countries such as the United States, Canada, and Australia do access residential aged care services (Chang et al., 2017; He et al., 2021; Koehn et al., 2018; Low & LoGiudice, 2018).

Recognizing the needs and preferences of individuals is fundamental in the provision of quality palliative care (World Health Organization, 2020). Several initiatives have been promoted to ensure that a person with a life-limiting illness is provided with the opportunity to communicate their values, wishes, and care preferences. Advanced care planning (ACP) is one such initiative that has been used (Fliedner et al., 2021). An international Delphi panel defines ACP as “a process that supports adults at any age or stage of health in understanding and sharing their personal values, life goals, and preferences regarding future medical care” (Sudore et al., 2017, p. 826). Completion of ACP is paramount with the increased presence of chronic conditions and cognitive impairment among older people in RACFs (or equivalent; AIHW, 2023b; Fagundes et al., 2021). The presentation of people greater than 85 years of age coupled with chronic conditions or impaired cognition may impact a resident’s capacity to make decisions and communicate care preferences (Sævareid et al., 2019). In Australia, considerable focus on ACP exists in the delivery of aged care services with an explicit requirement articulated in the Aged Care Quality Standards (Standard 2). This standard requires providers to comply with their obligations in relation to ACP and end-of-life planning (Aged Care Quality and Safety Commission, n.d.).

### ACP Among People of Chinese Ethnicity

Cultural values and beliefs influence a person’s perspective and attitude toward palliative care (Gerber et al., 2024; Six et al., 2023), including ACP engagement (McDermott & Selman, 2018). For people of Chinese ethnicity, findings from literature reviews reveal that they valued collective, family decision-making (Cheng et al., 2023; Pun et al., 2023, 2024) and thus could play a passive role in decisions related to ACP (He et al., 2021). Palliative and end-of-life decisions were predominantly made by the family and physicians (He et al., 2021; Pun et al., 2023, 2024). In a review by Jia et al. (2020), the authors make the point that contrary to the views about Chinese culture as a barrier to ACP completion (Cheung et al., 2020; Jiao & Hussin, 2020); Chinese people were not opposed to ACP discussions where a culturally sensitive approach was used. Findings from studies conducted in the community settings (Dhingra et al., 2020; Lee et al., 2017; Yap et al., 2018) similarly highlight that poor ACP uptake may be due to the lack of knowledge and awareness on the value of ACP completion rather than on culturally based reasons alone. From studies conducted in the United States, findings show that while most of the Chinese American participants had limited knowledge about ACP, they were open to discussion or had a positive attitude toward ACP completion (Dhingra et al., 2020; Lee et al., 2017). An Australian study (Yap et al., 2018) likewise found that while there were misconceptions and low awareness about ACP among older Chinese participants impacting ACP completion, the participants were open to conversations regarding future medical and end-of-life care.

In the RACF context (or equivalent), there is limited research around the ACP process and engagement of older Chinese people (He et al., 2021). In Australia, the RACF provider’s role includes initiating conversations on admission regarding ACP, verifying the presence of a substitute decision-maker, and ensuring that an ACP which reflects the person’s values and preferences is created (Advance Care Planning Australia, 2023). Written ACP documentation occurs in various forms including an advanced directive, advanced health directive, advance personal plan, medical direction and orders such as do not resuscitate and do not hospitalize (palliAGED, 2022). Contrary to the findings of some studies conducted in RACFs (or equivalent) in the United States (Manu et al., 2017; Resnick et al., 2009) and Europe (Kastbom et al., 2022) indicating a completion rate of advanced directives greater than 60%, evidence in Australian RACFs suggests a substantially lower prevalence of between 20% and 50% (Detering et al., 2019, 2021). Given the value of ACP in older people’s care in RACFs and that people of Chinese ethnicity are among the largest culturally and linguistically diverse (CALD) group worldwide (International Organization for Migration, 2022) including in Australia (Australian Bureau of Statistics, 2022), further research is needed that explores the uptake and documentation of ACP among older Chinese in the RACF context. A retrospective study of ACP in an Australian RACF will enable insights into the extent to which the ACP documentation considers the person’s cultural needs and preferences and their involvement in care planning to be known.

### Aim of the Present Study

This study aims to explore the presence or absence of ACP documentation in Australian RACFs with evidence of the older person who identify as Chinese ethnicity and the family’s involvement specific to their cultural needs. The study will answer the following questions: (a) What proportion of older people, who identify as Chinese ethnicity in RACFs have ACP documentation? (b) What evidence is provided in the documentation which conveys the older person, and their family are involved in the ACP process? and (c) What documentation is related to their cultural needs and preferences?

## Method

### Study Design, Setting, and Sample

A retrospective review of ACP was undertaken. The setting was RACFs in metropolitan areas of New South Wales, Australia. In New South Wales, there is no specific ACP form recommended. Two sites participated with a total of 201 beds. The sample included older people who identify as Chinese ethnicity as documented in the person’s demographic profile. Older people’s documentation was included if they: (a) identify as Chinese ethnicity; (b) had a diagnosis of a life-limiting illness; and (c) were a resident in one of the two RACFs during the data collection period.

### Data Source and Data Collection

Prior to the commencement of the study, the first author discussed ACP documentation in the participating RACFs with a clinical leadership staff member. In the participating RACFs, ACP was primarily documented in the Advanced Health Directive; however, documentation related to ACP was also included in the Multidisciplinary Case Conference which was conducted regularly with residents and their family or representative.

Staff members were invited by the organization to be assigned a role in the data collection. The staff were given an option of their preferred data collection method: (a) retrieve and de-identify the documents and send to the first author via email or (b) complete a data extraction template. The first and second authors met with staff and discussed the research project, eligibility criteria for older people’s documentation to be included, the process involved, and their respective roles and options with data collection. The first author provided staff with written instructions for each data collection method, and staff were given time to decide which method was preferable for them. Data were collected from March to June 2023.

### Data Extraction

A data extraction template was developed by the first author. The template contained three Microsoft Excel spreadsheets: (a) Advanced Health Directive; (b) Multidisciplinary Case Conference; and (c) Other documents where ACP documentation was included. Data were extracted from the time of the older person’s admission to the residential aged care facility. Following confirmation of Chinese ethnicity, demographic data collected were the older person’s age, medical diagnosis, and date of admission to the RACF. For the Advanced Health Directive, data extracted were date that the ACP was completed, duration of the ACP implementation, presence of the older person and their family at the ACP discussion, level of care received by the older person, and preferred care or directives. For the Multidisciplinary Case Conference, data extracted were date of the conference, presence of the older person and their family/representative, presence of people representing the facility, and care issues discussed in relation to ACP and cultural needs. Where an older person has another documentation which includes ACP, data extracted include the name of the document, date completed, and documentation in relation to cultural needs and preferences for palliative care.

### Statistical Methods

Descriptive statistics were undertaken to calculate proportions. The proportion of residential aged care residents with ACP documentation was calculated using the number of residents who identify as Chinese ethnicity as the denominator. Consultation with a statistician was done prior to the study. Due to the exploratory nature of the study, with no planned statistical tests of hypothesis, no formal power calculation was performed.

### Ethical Approval

The study was approved by the University. Organizational consent was obtained from the participating RACFs. As the RACF staff were involved either in the retrieval and de-identification of documents or in the extraction of information into the template, information accessed by the researchers and the statistician was de-identifiable.

## Results

From March to June 2023, a total of 31 residents met the eligibility criteria across two RACFs, equivalent to 15% of the residents from the two facilities. Chinese residents’ demographic characteristics are shown in Table 1. The Chinese residents’ mean age was 84.3 (±9.7) years and for 42% of residents the length of stay in the RACF was less than 12 months. Almost all the residents (*n* = 30, 97%) had two or more diagnosed chronic illnesses including dementia (*n* = 23, 74%). Musculoskeletal diseases and cerebrovascular diseases were the most common comorbid diagnoses. The other illnesses affecting about a third of the residents were type 2 diabetes mellitus, gastrointestinal disorders, and mental health problems.

**Table 1.** Demographic and Clinical Characteristics of Older Chinese Residents (n = 31).

Demographics	*n*	%^a^
Age, *M* (*SD*)	84.3 (9.7)	
Length of stay in the facility		
• 0–12 months	13	42
• 1–2 years	7	23
• 2 years	10	32
• No data	1	3.2
Diagnoses^b^		
• Dementia	23	74
• Cardiovascular diseases	12	39
• Diabetes mellitus	11	35
• Cerebrovascular diseases	13	42
• Musculoskeletal diseases	19	61
Number of medical diagnoses		
• 1	1	3.2
• 2 or more	30	97

a Percentages may not add up to 100 due to rounding.^b^ Residents may have more than one diagnosis.

### ACP Documentation With the Older Chinese and Their Family

An advanced health directive was completed for 90% of the residents (*n* = 28). Health professionals documented as involved in the discussion of ACP were the general practitioner and registered nurse. Of those completed, the presence of the resident (*n* = 26, 93%) and their representative (*n* = 24, 86%) were documented. Resident’s representative included their spouse, children, grandchildren, and friend.

Directives in relation to preferred medical care were clearly documented including cardiopulmonary resuscitation and level of care. All the residents with an advanced health directive had documentation regarding cardiopulmonary resuscitation, with more than half documented as not for cardiopulmonary resuscitation (*n* = 16, 57%). Half of the residents (*n* = 14, 50%) were classified as receiving limited care where hospital transfers, intravenous therapy, and antibiotics were to be considered only if appropriate. More than one third of the residents (*n* = 10, 36%) were classified on palliative care, thus receiving no hospitalizations, diagnostic testing, or treatments except for comfort measures. Other information included in the advanced health directive was the resident’s dietary status, with most residents (*n* = 20, 71%) on a regular diet. Most of the advanced health directives duration of implementation was indefinite (*n* = 19, 68%) as shown in Table 2.

**Table 2.** Documentation of Advanced Healthcare Directive in Residential Aged Care Facilities.

Advanced healthcare directive completion	*N*	%^a^
• Yes • No	28 3	90 9.7
Advanced healthcare directive variables	*n* (% out of 28)	
Competence^b^ (resident)		
• Yes	3	11
• No	22	79
• Missing data	3	11
Resident present		
• Yes	26	93
• Missing data	2	7.1
Resident’s representative present		
• Yes	24	86
• No	1	3.6
• Missing data	3	11
Cardiopulmonary resuscitation (CPR)		
• Person is for CPR	12	43
• Person is not for CPR	16	57
Level of care		
• Palliative^c^	10	36
• Limited^d^	14	50
• Intensive	2	7.1
• Missing data	2	7.1
Feeding		
• Basic^e^	20	71
• Supplement	4	14
• Supplement, or intravenous, or nasogastric	2	7.1
• Missing data	2	7.1
Implementation		
• 3 months	1	3.6
• 12 months	3	11
• Indefinite	19	68
• Until revoked	2	7.1
• Missing data	3	11

a Percentages may not add up to 100 due to rounding. ^b^ Competent means that the person has decision-making capacity. ^c^ Palliative care level is not for hospital transfer. Only measures that enhance comfort are to be provided. No diagnostic tests and treatments, such as antibiotics, are to be given unless these improve comfort. ^d^ Limited care level may be transferred to hospital and may receive intravenous therapy and antibiotics if appropriate. ^e^ Basic feeding refers to regular diet and no attempt to feed special diets, intravenous fluids, or tubes.

More than three quarters (*n* = 24, 77%) of the residents had a multidisciplinary case conference form completed. The presence of the resident (*n* = 19, 79%) and their representative (*n* = 23, 96%) were documented, with 92% represented by a family member. Five residents (21%) were documented as absent from the case conference. ACP was documented as being discussed in 96% of case conferences (*n* = 23). Table 3 summaries the documentation of multidisciplinary case conferences.

**Table 3.** Multidisciplinary Case Conference Documentation in Residential Aged Care.

Multidisciplinary case conference completion	*n*	%^a^
• Yes	24	77
• No	7	23
Multidisciplinary case conference variables	*n* (% out of 24)	
Resident present		
• Yes	19	79
• No	5	21
Persons representing the resident		
• Family member	22	92
• Friend	1	4.2
• Self (resident)	1	4.2
ACP discussed		
• Yes	23	96
• No	1	4.2
Social, religious, cultural needs, enjoyment of lifestyle activities discussed		
• Yes	14	58
• No	10	42
Spiritual support discussed		
• Yes	1	4.2
• No	23	96
Communication discussed		
• Yes	23	96
• No	1	4.2

a Percentages may not add up to 100 due to rounding.

### Documentation Related to Cultural Needs and Preferences

Data on cultural and spiritual needs and preferences were not included in the advanced health directive form for any of the residents as shown in Table 2. For the multidisciplinary case conference documentation, the other care issues discussed during the conference were social, religious, cultural needs, and lifestyle activities (*n* = 14, 58%) as shown in Table 3. Spiritual support was discussed with one resident only. Communication was documented as discussed in all but one record (*n* = 23, 96%). Details on the care issues discussed were not included in the completed form. One resident had another document completed in relation to ACP, which detailed the Buddhist way of end-of-life preparations. The document outlines perspectives from Buddhist scripture about the body’s experience at the verge of death and the person’s wishes prior to and during the end of life as shown in Table 4.

**Table 4.** Other Document: Buddhist Way of End-of-Life Preparations.

Period of illness or death trajectory	Actions from care staff that support the person’s wishes
Critically ill	• Not to touch or move the body. • Avoid external interruptions. • Chanting and playing of music until monks arrive
Unconscious breathing but no heart rate	• Avoid medical interventions. • Handle situation calmly, speak softly. • Call emergency contacts at appropriate time.
Impending death	• Contact Monk and Dharma friends. • Allow monks and chanting members to chant.
As soon as breathing stops	• Not to touch or move the body and the bed.
Within 8–10 hours after breathing stops	• Continue with recitation and chanting.
Funeral matters	• Done after 8–10 hours of recitation. • Body cleaning and dressing can be organized. Male staff preferred.

## Discussion

This study aimed to describe documentation of ACP including evidence of the older person and family involvement related to the cultural needs of older people who identify as Chinese ethnicity in Australian RACFs (or equivalent). To our knowledge, this is the first study that reports findings regarding ACP documentation among people of Chinese ethnicity in Australian RACFs (or equivalent). Overall, nine out of 10 residents of Chinese ethnicity in the participating facilities had ACP documentation completed. The presence of the resident and their representative and medical care directives were included in the documentation. However, specific details on the provision of palliative care that considers the person’s cultural needs and preferences were limited. Demographics of the older people in this study align with existing evidence on the prevalence of complex health conditions and dementia among the older population in an RACF (Fagundes et al., 2021; Kastbom et al., 2022; Muszalik et al., 2021).

### ACP Completion With Older Chinese and Their Family

Contrary to the literature revealing low ACP engagement among people from CALD backgrounds (Hong et al., 2018; McDermott & Selman, 2018) and low completion rate of advanced care directives in Australian RACFs (Advance Care Planning Australia, 2023; Detering et al., 2019, 2021), findings from this study show that ACP was mostly completed with the older Chinese residents and their family. The presence of the older Chinese residents and their family was included in the ACP documentation. The improved completion of ACP among the residents included in this study could be attributed to an increasing interest in and awareness of ACP among older people in the residential aged care settings. In recent years, several studies have explored ACP in RACFs (or equivalent; Buck et al., 2019; Lam et al., 2018; Martin et al., 2016; Ng et al., 2022; Tsai et al., 2017). Findings from these studies shed light on barriers and facilitators to the ACP process (Buck et al., 2019; Lam et al., 2018; Ng et al., 2022) and effects of ACP completion on residents in aged care facilities (Martin et al., 2016; Tsai et al., 2017).

In Australia, a Royal Commission into Aged Care Quality and Safety was established in 2018 to investigate the quality of aged care service provision (Commonwealth of Australia, 2021). The Royal Commission into Aged Care Quality and Safety (2019) noted the limited uptake of ACP in aged care and highlighted the important role of aged care providers in improving its uptake and implementation. Issues uncovered by the Royal Commission into Aged Care Quality and Safety (2021a) included substandard palliative care provision, which prompted an urgent review of palliative care practices in RACFs. As there were no further published studies on ACP completion since the final report and recommendations of the Royal Commission into Aged Care Quality and Safety (2021b) were published, it is not known if there is an overall improvement with ACP uptake in the general older people population in Australian RACFs.

### Documentation of Preferences for Future Care

Except for one resident with a Buddhist end-of-life preparation document, the ACP-related information documented for the residents in this study was restricted to that required in the advanced health directive and multidisciplinary case conference forms used by the participating RACFs. Findings from this study show that there were clear directives regarding preferred future medical care including resuscitation, hospitalization, and treatments. Much of the literature and guidelines around ACP documentation highlight that discussion of the medical treatment plan is a critical element of ACP (Rietjens et al., 2021). Documentation of specific medical treatments in the ACP is associated with positive outcomes including increased compliance with end-of-life wishes, satisfaction with care, improved communication, reduced hospital admission, and use of unwanted life-sustaining treatments (Brinkman-Stoppelenburg et al., 2014; Kastbom et al., 2022; Martin et al., 2016).

While there was good documentation of medical care preferences, this was not the case for other domains such as cultural and spiritual needs or preferences. Culture and spirituality-related documentation was not included in the primary ACP document. From the multidisciplinary case conference, documentation of information related to culture and spirituality mainly conveyed that a discussion occurred regarding these domains, but there were no specific details provided that could be used as a guide in care provision. Discussion and documentation of personal life goals and values that may not be related to medical treatments are recommended both in the Australian and international contexts (NSW Ministry of Health, 2022; Rietjens et al., 2021). For people from CALD backgrounds, considerations for cultural and spiritual aspects of care are particularly important (Gerber et al., 2024). Findings from previous research have shown the influence of culture and ethnicity on a person’s preferences for palliative and end-of-life care. In a scoping review on end-of-life care for older first generation migrants, Gerber et al. (2024) note the influence of culture and religion on end-of-life discussions such as in terms of the degree of family member’s involvement, dying as a taboo subject for some cultures, and preferred level of disclosure. In a study involving four culturally diverse groups in Australia, Ohr et al. (2017) found the varying influence of culture and ethnicity on death and dying and ACP. For example, the Eastern European and Asia and Pacific groups held stronger belief that death should be avoided at all costs compared to the Anglo-Celtic and Mediterranean groups (Ohr et al., 2017). In a systematic review of spirituality in palliative care from an Indian perspective, Gielen et al. (2016) found that their unwavering faith in God’s healing power influences their engagement in religious practices including prayers, songs, medication, and yoga, among others.

Despite acculturation among Chinese people in Western countries, they continue to maintain their cultural identity, values, and behaviors (Mao et al., 2020). The Buddhist way end-of-life preparation document for one resident in this study aligns with evidence from literature showing the influence of cultural and religious traditions among Chinese people in ACP and end-of-life care practices. In a qualitative study exploring end-of-life beliefs, values, and practices among Chinese women living in England (Fang et al., 2015), participants mentioned several practices which were underpinned by their cultural and religious traditions. For example, when a person dies, they burn incense to respect and honor the deceased, burn earthy materials such as paper money as a symbol of transferring earthly wealth to after life, and involve a Taoist priest to conduct rituals (Fang et al., 2015). In an article on death and dying among people of Chinese ethnicity, Hsu et al. (2009) similarly noted several practices which are nested within Chinese philosophies and religions. Death and dying practices cited by Hsu et al. (2009) include ancestor worship which fosters a continued connection of the deceased person’s spirit with life on earth, religious traditions with various perspectives and practices on death and dying, and use of traditional Chinese medicine for management of symptoms of the dying person. Including information on cultural values and beliefs related to future care preferences in the ACP documentation for RACF residents of Chinese ethnicity is therefore very important.

### The Importance of Comprehensive ACP Documentation

Provision of care that acknowledges the person’s cultural identity could be a challenge in an RACF (or equivalent) due to contextual, organizational, and workforce-related factors (Gaviola et al., 2023). Compounding the challenges in care provision are the complex health conditions of older people in residential aged care with a prevalence of dementia diagnosis (Fagundes et al., 2021; Kastbom et al., 2022) which affects the person’s ability to communicate their wishes and preferences. Thus, the need for a comprehensively documented ACP is inarguably critical in this setting. A documented ACP helps ensure that information is available regarding the person’s unwanted treatment and wishes, views, and values that inform future health care (Carter et al., 2015; Ng et al., 2022; Sævareid et al., 2019). While ACP is not restricted to a written format, a written document strengthens its validity and applicability (Carter et al., 2015) and the alignment of care provision with the person’s preferences (Sudore et al., 2017). Given the diverse demographics of older people in residential aged care, key recommendations for ACP encompass diversity; hence, inclusion of information related to engagement with consumers from CALD backgrounds is warranted (Advance Care Planning Australia, 2023). It is important to ensure the presence of a documented ACP which does not only capture the preferred medical treatments but also the cultural and spiritual domains of care, such as the case for the Buddhist end-of-life document of one resident in this study.

### Limitations

This study has some limitations including the small sample size, the source of ACP documentation, and the concept of the older Chinese’s presence versus involvement during the ACP process. Recent data show that only about 19% of older people living in Australian RACFs are from CALD backgrounds (Department of Health and Aged Care, 2022). While people of Chinese ethnicity may make up the majority of CALD residents, there is no information on the specific cultural background of older people in residential aged care that could be used as a reference. The source of ACP documentation was mainly advanced health directive forms and the multidisciplinary case conference; nearly all residents of Chinese ethnicity had an ACP. However, these forms did not specifically require detailed information on cultural needs. RACF staff were asked to retrieve any other documents with information on ACP; however, there may have been other documentation of ACP information that were missed. A standardized approach was used to retrieve information from records; however, relevant detail may have been missed. Importantly, the proportion of residents who were asked about specific cultural needs could not be determined. Finally, while the ACP documentation indicates the presence of the older person, how active they were in the ACP decision-making was unable to be determined.

### Implications for Practice and Research

The findings of this study are a starting point for addressing the gaps around culturally sensitive ACP documentation for people of Chinese ethnicity and, to a larger extent, people from other culturally diverse backgrounds. Further research exploring the culture-specific elements of ACP that are relevant to people of Chinese ethnicity including cultural values and beliefs related to death and dying would inform this documentation. For RACFs (or equivalent), a review of existing ACP process and documentation could be undertaken to determine an optimal way of embedding culture-specific information and to develop a culturally sensitive ACP that considers the diverse needs and preferences of people of Chinese ethnicity.

## Conclusions

An advanced care plan was completed for almost all older Chinese aged care residents in this retrospective review. The presence of the resident and family and preferred or unwanted medical care were well documented; however; documentation on cultural aspects of care was limited. Specific details on the provision of palliative care that considers the person’s cultural needs and preferences would have been valuable. From the findings of this study and with the limited literature on what a culturally sensitive ACP might look like for people of Chinese ethnicity, there is a need for further studies that explore ACP in the context of this population’s diverse cultural background and guidelines on culturally responsive ACP for providers of care to the older people.
